# Modulation of amygdala activity for emotional faces due to botulinum toxin type A injections that prevent frowning

**DOI:** 10.1038/s41598-023-29280-x

**Published:** 2023-02-27

**Authors:** Shauna Stark, Craig Stark, Brian Wong, Mitchell F. Brin

**Affiliations:** 1 Department of Neurobiology and Behavior, University of California Irvine, Irvine, USA; 2 Department of Otolaryngology-Head and Neck Surgery, University of California Irvine, Irvine, USA; 3 Department of Neurology, University of California Irvine, Irvine, USA; 4 Research and Development, BOTOX® and Neurotoxins, Allergan, an AbbVie Company, 2525 Dupont Drive, T2-3, Irvine, CA 92623-9534 USA

**Keywords:** Neuroscience, Physiology, Psychology, Medical research, Neurology

## Abstract

According to the facial feedback hypothesis, when we see an angry or happy face, we contract or flex the relevant muscles to recreate the expression to assist in identifying and experiencing the emotion reflected. We investigated the facial feedback hypothesis by using botulinum toxin type A (onabotulinumtoxinA; onabotA) injections to induce temporary paralysis in the glabellar muscles (responsible for frowning) and measured functional brain activity during the processing of emotional faces. Ten females viewed pictures of happy and angry faces during two functional magnetic resonance imaging (fMRI) scan sessions: one prior (Pre) to onabotA and one following (Active) onabotA injections. We found Pre vs. Active onabotA modulation of activity in the amygdala for both happy and angry faces, as well as modulation of activity in the fusiform gyrus for happy faces. Consistent with our predictions, preventing frowning through inhibition of glabellar muscle contraction altered amygdala processing for emotional faces. The modulation of amygdala and fusiform gyrus activity following onabotA may reflect compensatory processes in a neuroanatomical circuit involved in emotional processing that is engaged when facial feedback is impaired. These data contribute to a growing literature suggesting that inhibition of glabellar muscle contraction alters neural activity for emotional processing.

Clinical Trials.gov registration number: NCT03373162.

## Introduction

The “facial feedback hypothesis” proposes that muscle memory in the face interacts with emotional regions of the brain, particularly the amygdala, and that this signaling is bidirectional^1,2^. Specifically, the model proposes that afferent feedback signals from facial muscles influence how we process and experience emotion^3^, while the efferent connections from the brain are responsible for producing emotional facial expressions. For example, the corrugator supercillii, a component of the glabellar muscles (the “frown muscles” between the eyebrows) has been associated with creating an angry expression^4^ and is engaged upon viewing photos of angry facial expressions^5,6^. Similarly, corrugator muscle movement associated with negative pictures is consistent with the modulation of amygdala activity and subjective ratings of negative valence^7,8^.

To more directly evaluate the link between corrugator muscle activity and processing of negative emotions, several studies have attempted to inhibit those muscles via injections of botulinum toxin A (BoNTA). BoNTA injections cause a temporary relaxation of the muscles at the injection site by inhibiting the release of acetylcholine at motor nerve endings^9^. Thus, BoNTA injections in the glabellar region temporarily block the afferent feedback signals from these muscles, allowing for investigation of emotional processing while deprived of facial muscle feedback. Preventing a facial frown through the use of BoNTA injections has been shown to influence the subjective experience of emotional responses to video clips^10^ and emotional language processing^11^.

Similarly, several studies have suggested that peripheral BoNTA injections in the region of the glabellar muscles may be effective in treating major depression^12–17^, suggesting a possible role of neuromodulation injections in altering emotional processing. In addition, inverse frequency analyses of the FDA Adverse Event Reporting System (FAERS) database found that depression was reported at a significantly lower rate among individuals treated with BoNTs compared with a control group of patients who received any medication for depression (N > 8 million adverse event reports)^18^ and compared with those who received other treatments for a variety of conditions (N > 13 million reports; e.g., cosmetic, chronic migraine, spasticity)^19^.

In addition, BoNTA injections in the glabellar region have been shown to modulate amygdala activity in response to the imitation of emotional expressions^20^ and during viewing of emotional faces^21^. These results support a growing body of research suggesting that peripheral motor function is intrinsic to the perception of emotion and may have broader impacts on cognitive processing. These studies also support a key tenant of the facial feedback hypothesis: the inability to draw the eyebrows down into a frown may alter the emotional processing in the brain and in behavior.

Here, our aim was to better understand the effect of BoNTA (as onabotulinumtoxinA; onabotA) on functional activity in the brain during the processing of emotional faces. We first sought to test the hypothesis that onabotA injections would alter emotional-related activity in the amygdala, as shown by others^21^. We then extended this work by evaluating regions outside of the amygdala to determine if onabotA alters facial processing as part of the facial feedback mechanism.

## Methods

**Figure 1:**
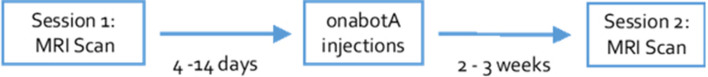
Schematic of study design.

This pilot study had a pre- post design: the first MRI scan session was conducted 4–14 days prior to the onabotA injection session and the second was conducted 13–23 days post-injection (Fig. 1). Dosing studies have shown that onabotA is fully active at 14 days and still maximally sustained at 28 days^22^. In order to maximize our resources and have as much statistical power as possible with our sample size, we relied upon a pre-post study design to evaluate the effect of onabotA injections.

### Participants

Ten healthy, right-handed females (mean age = 36.4; range = 33–40 years old) with no history of using botulinum toxin participated in the study. We restricted enrollment to only females because emotional responsivity varies for males and females^23–25^ and we wanted to reduce variability given our small sample size. Prior to enrollment, participants were screened and excluded for the presence of neurological or psychiatric conditions and the presence of any risk factors for MRI. Participants were recruited from the University of California at Irvine (UCI) and the surrounding community via e-mail blasts, flyers, social media, and word-of-mouth. They provided written consent in compliance with the UCI Institutional Review Board and received $100 compensation for 2 MRI scans and free cosmetic onabotA injections. This study was approved by the UCI Institutional Review Board and was conducted in accordance with the Declaration of Helsinki.

### OnabotA injections

**Figure 2﻿:**
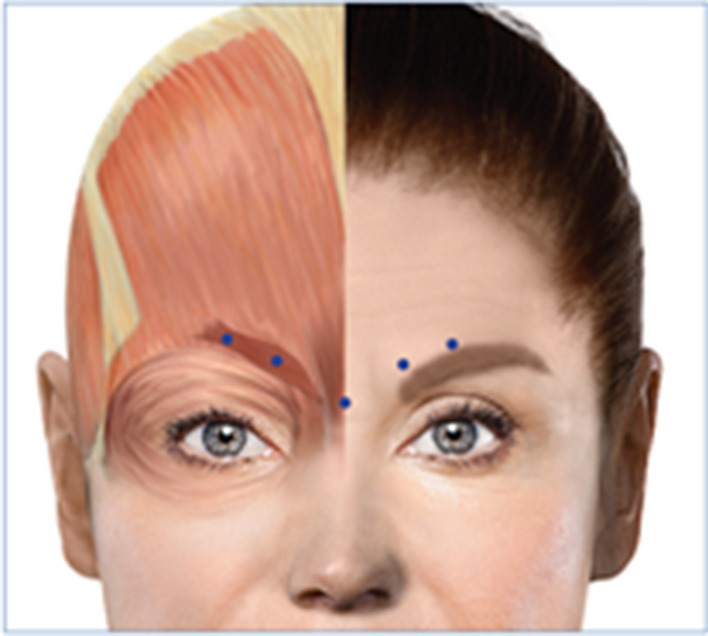
OnabotulinumtoxinA injection sites. This figure was modified from Blumenfeld and colleagues^60^ under a Creative Commons license (CC BY-NC 4.0).

Participants received injections of onabotA in the glabellar region from one investigator (BW). Each participant received 20 units total, which was diluted as 100 units onabotA/2.5 ccs non-preserved injectable saline for a total volume per injection of 4 units/0.1 cc. This dose was distributed across the injection sites and is adequate to inactivate the glabellar region in naïve participants^22^. During a single session, participants were injected five times (4 U each) with onabotA: twice in the corrugator supercilia on each side (located at the medial end of each eyebrow) and once in the procerus (the vertical midline muscle that pulls the medial ends of the eyebrow downwards) (Fig. 2). At their post-scan visit, all participants reported noticing a physical change in sensation and difficulty pulling their eyebrows together, demonstrating effectiveness of the onabotA injections.

### fMRI task

Given the relatively sparse literature in the area, we chose to follow a similar protocol to Kim and colleagues^21^, in which photos of angry and happy facial expressions were presented for 50 ms or 1000 ms, followed by a black and white patterned mask, presented for 250 ms (serving as a retinal wipe), and a 1500 ms intertrial interval. During each trial, participants used a button box to indicate whether they thought each of the faces was pleasant or unpleasant to ensure that participants were evaluating the emotional expression of the face. The stimuli were from standardized databases (NimStim Face Stimulus Set) with established validity ratings for the displayed emotion^26,27^. Unlike the prior work, a visual discrimination task (32 trials per run) was randomly intermixed with face trials (84 faces per run), in which participants were instructed to identify which of two squares on the screen was the brightest^28^. This task served as a baseline for the fMRI analysis, which does not engage limbic structures, making it an ideal control condition. Four separate versions of the task were created and counterbalanced across pre- and post-scans per subject, with two runs per scan session, each totaling approximately 5 min.

### fMRI protocol

Each scanning session lasted approximately one hour. All participants were scanned at the Facility for Brain Research (FIBRE) at the University of California, Irvine, using a Siemens Prisma 3.0 T MRI with a 32-channel head coil. Functional images were acquired using an echo-planar T_2_*-weighted imaging sequence. Each volume consisted of 64 interleaved 2.1 mm-thick axial slices with a slice acceleration factor of 8 (echo time (TE) = 34 ms, repetition time (TR) = 1500 ms, field of view (FOV) = 202 mm, flip angle = 75°, voxel size = 2.1 × 2.1 × 2.1 mm). Anatomical T1-weighted images were collected using magnetization-prepared rapid gradient-echo (MP-RAGE) imaging, with 320 interleaved 0.8 mm-thick axial slices with an acceleration factor of 3 (TE = 2.4 ms, TR = 2300 ms, FOV = 256 mm, flip angle = 8°, voxel size = 0.8 × 0.8 × 0.8 mm). We also collected a series of magnetic resonance spectroscopy scans, but that data is not included in this report.

### fMRI preprocessing

Results included in this manuscript come from preprocessing performed using FMRIPREP^30^ [RRID:SCR_016216] version #1.0.0-rc5, a Nipype^31^ [RRID:SCR_002502] based tool. Each T1w (T1-weighted) volume was corrected for INU (intensity non-uniformity) using N4BiasFieldCorrection v2.1.0^33^ and skull-stripped using antsBrainExtraction.sh v2.1.0 (using the OASIS template). Brain surfaces were reconstructed using recon-all from FreeSurfer v6.0.1^34^ [RRID:SCR_001847], and the brain mask estimated previously was refined with a custom variation of the method to reconcile ANTs-derived and FreeSurfer-derived segmentations of the cortical gray-matter of Mindboggle^35^ [RRID:SCR_002438]. Spatial normalization to the ICBM 152 Nonlinear Asymmetrical template version 2009c^36^ [RRID:SCR_008796] was performed through nonlinear registration with the antsRegistration tool of ANTs v2.1.0^37^ [RRID:SCR_004757], using brain-extracted versions of both T1w volume and template. Brain tissue segmentation of cerebrospinal fluid (CSF), white-matter (WM) and gray-matter (GM) was performed on the brain-extracted T1w using *fast* ^38^ (FSL v5.0.9, RRID:SCR_002823).

Functional data was slice time corrected using 3dTshift from AFNI v16.2.07^39^ and motion corrected using *mcflirt*^40^ (FSL v5.0.9). "Fieldmap-less" distortion correction was performed by co-registering the functional image to the same-subject T1w image with intensity inverted^41,42^ constrained with an average fieldmap template^43^, implemented with antsRegistration^36^ (ANTs). This was followed by co-registration to the corresponding T1w using boundary-based registration^44^ with 9 degrees of freedom, using *bbregister* (FreeSurfer v6.0.1). Motion correcting transformations, field distortion correcting warp, BOLD-to-T1w transformation and T1w-to-template (MNI) warp were concatenated and applied in a single step using antsApplyTransforms^36^ (ANTs v2.1.0) using Lanczos interpolation.

Physiological noise regressors were extracted applying CompCor^45^. Principal components were estimated for the two CompCor variants: temporal (tCompCor) and anatomical (aCompCor). A mask to exclude signal with cortical origin was obtained by eroding the brain mask, ensuring it only contained subcortical structures. For aCompCor, six components were calculated within the intersection of the subcortical mask and the union of CSF and WM masks calculated in T1w space, after their projection to the native space of each functional run. Frame-wise displacement^46^ was calculated for each functional run using the implementation of Nipype. Many internal operations of FMRIPREP use Nilearn^47^ [RRID:SCR_001362], principally within the BOLD-processing workflow. For more details of the pipeline see https://fmriprep.readthedocs.io/en/latest/workflows.html.

Using 3dDeconvolve (AFNI), we used a deconvolution approach based on multiple linear regression to analyze each functional voxel based on happy and angry events without an assumed hemodynamic response. The hemodynamic responses for each event of interest was estimated using 13 time-shifted tent functions, estimating the BOLD activity from 0 to 18 s after trial onset. In addition to these, nuisance regressors derived from *fmriprep* were included for: white matter signal, global signal, the first two aCompCorr components, framewise displacement, and the canonical six motion vectors (translation and rotation for each axis). For our trial types of interest, the resulting time-shifted beta coefficients represent activity versus the perceptual baseline for each regressor of interest at a given time point in each voxel.

For our a priori hypotheses, we conducted an anatomical region of interest (ROI) analysis by averaging the beta coefficients for two ROIs: right and left amygdala (based on FreeSurfer definitions of the amygdala). For our exploratory whole-brain analyses, we blurred the EPI data by 4 mm and used 3dMVM (AFNI) for a group-level ANOVA with Session (Pre vs Active) by Emotion (Happy vs Angry). As our primary interest was a modulation in emotional activity across session, but as our power was low, we chose a two-step approach. First, we identified regions showing a potential difference in activity based on emotion by a liberal threshold (main effect of emotion *p* < 0.05 voxelwise, 100 contiguous voxels). We then averaged activity within these clusters to enhance signal to noise and performed the orthogonal Pre-onabotA versus Active-onabotA test on this cluster-averaged activity with a final alpha threshold of *p* < 0.05.

## Results

### Behavioral results

**Figure 3:**
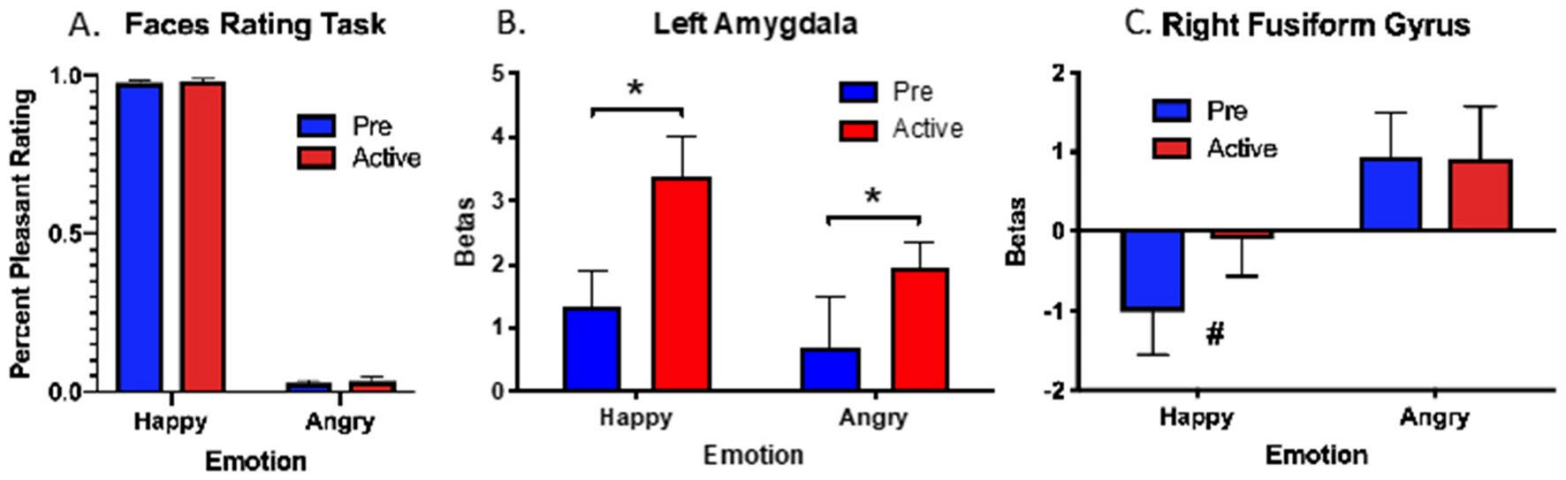
(**A**) Mean of percent pleasantness rating for happy and angry faces. Pleasantness ratings were higher for happy than angry faces and performance did not differ for ONABOTA-Pre and ONABOTA-Active injection. (**B**) In our a priori anatomical analysis of the amygdalae, we observed pre-post BOLD increase in left amygdala for both happy and angry faces. C) Likewise, we observed a BOLD increase in right fusiform gyrus for happy faces following the ONABOTA injection. Error bars show standard error of the mean. * indicates *p* < 0.05, # indicates *p* = 0.06.

We calculated the percent pleasantness rating for angry and happy faces. One participant’s data were excluded as her ratings scored more than 2 standard deviations outside the mean of the group. A 2 × 2 repeated-measures ANOVA with Session (Pre-onabotA vs Active-onabotA) by Emotion (Happy vs Angry) as variables revealed a significant main effect of Emotion (F(1,8) = 4340, *p* < 0.0001), with greater pleasantness ratings for happy faces (97%) than angry faces (3.5%). As expected, there was no main effect of session and no interaction, indicating no effect of onabotA on this simple emotional rating task (Fig. 3A). We chose to combine the stimulus presentation times as there were no differences in pleasantness ratings for the 50 ms or 1000 ms conditions.

### A priori anatomical results

We entered the average fMRI activity from the a priori amygdala ROIs for each subject into 2 × 2 repeated-measures ANOVA with Session (Pre-onabotA vs Active-onabotA) by Emotion (Happy vs Angry) as variables. Based on a 2 × 2 ANOVA, the left amygdala (Fig. 3B) showed a main effect of Session with greater fMRI activity in the Active-onabotA than Pre-onabotA condition (F(1,9) = 6.8, *p* < 0.05). We also found some evidence for a main effect of Emotion (Happy > Angry, F(1,9) = 4.4, *p* = 0.07), but no reliable evidence for an interaction (F(1,9) = 2.7, *p* = 0.14). In contrast, while the right amygdala showed a main effect of emotion (Happy > Angry, F(1,9) = 5.5, *p* < 0.05), it showed no effect of Session (F(1,9) = 0.16; *p* = 0.70) or interaction (F(1,9) = 1.5; *p* = 0.24).

### Whole-brain exploratory results

Next, we conducted a whole-brain exploratory analysis to determine if regions outside of the amygdala showed similar modulation of activity by onabotA. To mitigate statistical power loss in this whole-brain analysis, we first identified regions that showed a potential main effect of Emotion (Happy vs. Angry) collapsing across Session using an uncorrected threshold (*p* < 0.05, 100 contiguous voxels). These threshold parameters revealed activity localized to four regions: left fusiform gyrus, right fusiform gyrus, left inferior frontal gyrus, and right lingual gyrus. After identifying these regions as being sensitive to emotion, we asked the critical, orthogonal question of whether their activity changed as a function of treatment, by collapsing activity within the regions and subjecting them to a traditional ANOVAs with a final alpha of *p* < 0.05. Only, the right fusiform gyrus (Fig. 3C) showed greater fMRI activity in Active-onabotA than Pre-onabotA (F(1,9) = 63.2, *p* < 0.01), with this effect being largely driven by an increase in activity for happy faces (t(9) = 2.1, *p* = 0.06) in Active-onabotA.

## Discussion

**Figure 4:**
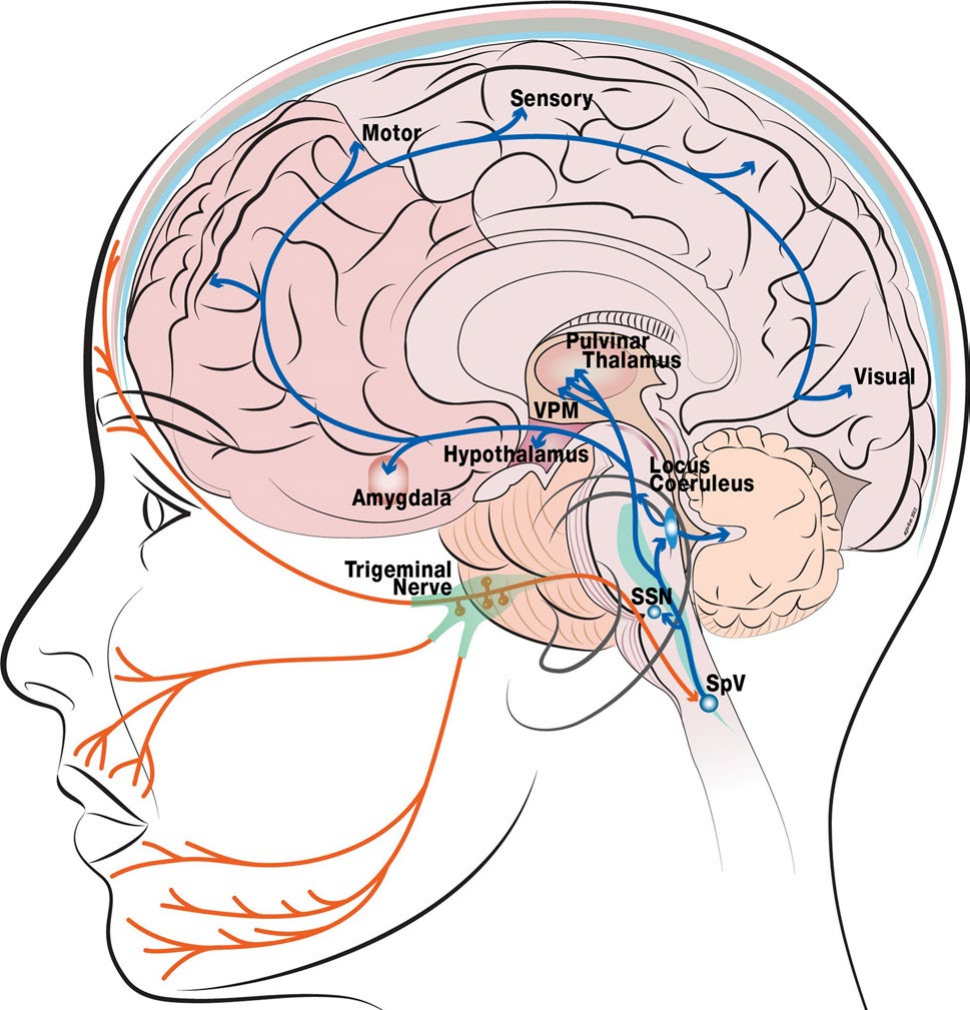
Neuroanatomical circuit involved in the processing of emotional faces. Orange lines represent the sensory trigeminal innervating the brain stem and synapsing on the trigeminal nucleus. The blue lines highlight the flow of information along key regions in the circuit. Sensory neurons in the trigeminal nucleus caudalis have reciprocal connections with sensory and limbic structures and are often monosynaptic^48,61–64^. These include trigemino-amygdala, trigemino-hypothalamus, trigemino-thalamus, and trigemino-locus coeruleus connections. Layers of the skull: white, scalp; pink, periosteum; grey, bone; blue, meninges (dura, arachnoid, pia); SpV: spinal tract of the trigeminal nucleus; SSN: superior salivary nucleus;VPM: ventral posteromedial nucleus. This figure was created in Adobe Illustrator^65^ (versions 2015 and 2021).

The facial feedback effect states that when we contract or flex the relevant muscles to create an emotional expression (e.g., happy or angry), it can assist in identifying and experiencing the emotion reflected, even in the absence of an emotional face as the stimulus. There is evidence that signaling between the emotional centers of the brain and facial muscles is bidirectional^1,2^, contributing to a neural circuit involved in the processing of emotions (Fig. 4). Corrugator muscle activity is sensed through facial nerves that innervate proprioceptive fibers of the optic branch of the trigeminal nerve. The mesencephalic trigeminal nucleus feeds into the locus coeruleus and amygdala^48^, which has direct connections with the prefrontal cortex^49^, both structures critical for emotional regulation^50^. The amygdala is responsive to emotional valence, often responding strongly to fear and arousal, but also when the specificity and differentiation of emotion has self-relevance or a strong relationship to one’s goals^51^. Thus, deactivation of the glabellar region can have a downstream effect in the neuroanatomical circuit involved in the processing of emotional faces.

In addition to the well-established modulation of amygdala activity by emotion, we found evidence consistent with that of prior studies by Kim and colleagues^21^, Hennenlotter and colleagues^20^, and Kruger and colleagues^52^, in that the amygdala’s responsiveness to emotion can be modulated by local facial chemodenervation. We do acknowledge differences in direction of effects but note differences in the contrasts and baselines across experiments. Given that the pre-treatment pattern differed in these experiments, it is clear that our participants treated the happy and angry faces differently. Amygdala activity has been observed for both happy and angry faces^53,54^, reinforcing the importance of this region for the processing of multiple emotions. Interestingly, we found an increase in amygdala activity for both happy and angry faces following onabotA injections, suggesting that blocking motor activity of the glabellar region affects both happy and angry expressions. Based on the facial feedback hypothesis, this net change in facial expression may reduce the internal experience of negative emotions and promote positive ones. Studies that further evaluate the expression of that emotion through behaviorally sensitive testing to determine if this neural signature represents changes in perception of both angry and happy facial expressions would be informative.

In addition to the amygdala, we observed a modulation of activity in the fusiform gyrus following onabotA injections. The fusiform gyrus (also known as Broadmann area 37) contains a region often labeled the fusiform face area because of its importance for processing faces and facial expressions^55^. Prosopagnosia patients have developmental or acquired dysfunction of the fusiform gyrus, which impairs their ability to recognize and discriminate among faces^56^. In addition to face recognition, the fusiform gyrus has also shown differential activity for emotional faces, including angry and happy emotions^57^, and interacts within a larger network of regions involved in emotional processing, including the amygdala^53^. Interestingly, we observed modulation of activity for happy faces and not angry ones in the fusiform gyrus. However, there is evidence that both frightened and happy expressions have elicited fusiform activity compared to neutral faces, suggesting a role for increased attention to emotional aspects of the faces, possible via the amygdala^58^. Our inability to detect differences in fusiform activity for angry faces is unclear. Exploration of a wider range of emotions to evaluate fusiform reactivity to emotional facial expressions may be informative.

We designed this study to parallel that of Kim and colleagues^21^, and there are some parallels in the results. In both the present study and in Kim and colleagues^21^, onabotA injections altered how the amygdala responded to emotional faces despite no effect on simple behavioral emotional ratings. However, there are several differences worth noting. Kim and colleagues^21^ implemented a Pre-onabotA, Active-onabotA, and washout (Post-onabotA) design, in contrast to our two (Pre- and Active-) time-points. They observed fMRI activity in the right amygdala in which Angry > Happy Pre-onabotA, a reversal to Happy > Angry during the Active-onabotA phase, and a return to Angry > Happy Post-onabotA. Here, we found left amygdala modulation and no significant findings in the right amygdala. Interestingly, the study by Hennenlotter^20^, found that imitation of angry faces increased activity in both the left and right amygdala, but that BoNTA blocked this increase in the left amygdala only. Thus, the hemispheric finding in Hennenlotter^20^ was the same as the present study, but the direction of the relationship was different (i.e., BoNTA blockade of amygdala activation vs. potentiation in the present study). In contrast to the present study, Hennenlotter and colleagues^20^ did not test happy faces (but tested angry and sad faces) and included separate groups of participants in their Pre- and Post-BoNTA groups.

The reason(s) for the hemispheric discrepancies are unclear but may be due to experimental design. The baseline contrast condition was different between the current study and that of Kim and colleagues^21^. Their baseline condition was uncontrolled (a mix of “surprised” faces and rest), making it impossible for them to reliably assess effects on emotional activity writ large. In the present data, we saw Happy ≥ Angry throughout. By virtue of having a controlled, non-emotional baseline (a challenging perceptual discrimination task) that does not modulate activity in limbic structures, we were able to look for overall shifts in emotion-related activity in the amygdala that Kim and colleagues^21^ could not. Here, we saw an increase in activity in the left amygdala post treatment regardless of which emotion was present. This difference in the baseline contrast may also account for the increase in activity in the left amygdala that we observe, whereas in the Kim et al. study, amygdala activity versus baseline cannot be determined from the data presented. The unstable baseline contrast by Kim and colleagues^21^ could also mask an overall increase in activity during Active-onabotA treatment or a selective increase in activity to surprised faces resulting in a relative decrease in activity for emotional faces. While the findings here do not invalidate other findings in the literature, they may reflect a more accurate modulation of both positive and negative emotional valence following deactivation of the glabellar region.

Each of these studies is relatively underpowered—though that is not unusual in functional imaging studies. Our results are based on a group of 10 participants, while Kim and colleagues^21^ reported on a group of only 7 participants and Hennenlotter^20^ included 38 participants. While there is some evidence for lateralization in amygdala activity for emotional faces^59^, future studies will be required to determine the source of this difference (including the task performed) and how relevant it might be for the facial feedback mechanism for emotional processing.

This study has several strengths and limitations. The sample size of the present study was small; larger studies will help increase the signal-to-noise ratio and improve generalizability. Additionally, the emotional faces task was limited to happy and angry faces. Use of a wider range of emotions (e.g., sad, surprised, scared) would address the generality of the amygdala response to other emotions. Finally, the present study enrolled healthy individuals. Studies of emotional modulation in clinically depressed individuals, already experiencing alterations in emotional processing, may reveal stronger effects of onabotA injections on emotion-related neural activity. Strengths of the present study include use of a controlled, non-emotional baseline that enabled us to assess overall shifts in emotion-related activity in the amygdala. We also conducted a whole-brain exploratory analysis to identify regions other than the amygdala that were modulated by onabotA, which led to the observation of significant effects in the fusiform gyrus.

## Conclusions

The present results provide additional evidence that neuromuscular feedback from creating an emotional expression can influence activity in two key regions for processing emotional faces: the amygdala and the fusiform gyrus. Inhibition of the glabellar region muscles prevented frowning and reduced the creation of smiling or happy expressions, resulting in alterations in amygdala activity for both happy and angry faces. The increase in amygdala activity may reflect compensatory processes during emotional processing that are engaged when facial feedback is modulated. While there remains much more to explore regarding the role of facial feedback on amygdala and fusiform gyrus activity, as well as on other regions involved in the neuroanatomical circuit for processing emotional faces, these data contribute to a growing body of literature suggesting that inhibition of facial muscles can alter neural activity for emotional processing.

## Data Availability

Data from this project, either in summary form or in raw form, will be made available upon request (contact corresponding author, Dr. Mitchell Brin) for researchers wishing to use the data for non-commercial purposes.
